# Lessons From India: A Narrative Review of Integrating Yoga Within the US Healthcare System

**DOI:** 10.7759/cureus.43466

**Published:** 2023-08-14

**Authors:** Puja Yatham, Supritha Chintamaneni, Sarah Stumbar

**Affiliations:** 1 Internal Medicine, Herbert Wertheim College of Medicine, Miami, USA; 2 General Medicine, Jagadguru Sri Shivarathreeshwara Medical College, Mysore, IND; 3 Family Medicine, Herbert Wertheim College of Medicine, Miami, USA

**Keywords:** holistic healthcare, preventive health, yoga benefits, integrative health, yoga, us healthcare, indian health system

## Abstract

The ancient practice of yoga has gained worldwide popularity as a way for people to improve their overall health and well-being. This manuscript reviews and examines the history of yoga, its physical and mental health benefits, its incorporation into the Indian healthcare system, and the public perception of yoga in India. Many initiatives for yoga exist, including promoting research on yoga, providing education and information on its benefits, and developing evidence-based standardized yoga therapy guidelines. With this in mind, this narrative review article explores the potential benefits of incorporating yoga into the United States (US) healthcare system and the possible challenges of doing so. It also provides valuable insights for policymakers and healthcare professionals.

## Introduction and background

Yoga is an essential component of India's healthcare system and is deeply rooted in Indian culture [1]. Its foundation is based on philosophies and spiritual practices that date back thousands of years to ancient India [2]. The word “yoga” is derived from Sanskrit and means “to unite,” which requires conceding the mind, body, and spirit [3]. The eight limbs of yoga, which include ethical conduct, physical postures, breath control, withdrawal of the senses, concentration, meditation, and oneness with the object of meditation, help achieve the goal [2,4].

Over the centuries, Yogic practices have been a continuous element in homes and ashrams across the Indian subcontinent. Mohenjo-Daro, an archaeological site in the Indus Valley, contains evidence of the earliest carvings for yoga dating back between 2,500 and 5,000 years ago [5]. In recognition of its importance, the country has facilitated the establishment of many universities dedicated to teaching and researching yoga [6]. There are various forms of yoga, including Bhakti (spiritual), Jnana (knowledge), and Karma (duty and action) [7].

Sage Patanjali, known as the father of yoga, played a vital role in cataloging Raja yoga, which includes mental discipline, controlled postures, conscious breathing, detachment, and meditation [8]. During the 10th century C.E., yogic practices called Tantra developed, which claimed to tap into the serpentine energy known as “Kundalini,” located at the base of the human spine. Originating between the 10th and 12th centuries C.E., Hatha yoga focuses on extreme psychophysical practices to channel inner energy. The modernization of yoga has created room for various combinations of Raja and Hatha yoga, emphasizing meditation, breathing, and postures [9].

As yoga's benefits have become increasingly evident over the years, numerous recommendations for incorporating yoga into healthcare systems have emerged, some of which are discussed in this narrative review. This review examines the history, physical and mental health benefits, and incorporation of yoga into the Indian healthcare system while casting light on the unique potential and challenges of integrating yoga into the US healthcare system. In our literature review, we have not encountered any articles addressing a comprehensive review of yoga benefits and its incorporation into a healthcare system. The goal of this narrative review is to offer valuable insights to policymakers and healthcare professionals in comprehending the potential benefits and challenges of integrating yoga into the US healthcare system by providing a holistic perspective on the subject.

## Review

Incorporation of yoga into healthcare in India

As early as the 1940s, the government of India set up various structures, including the Bhore Committee, Chopra Committee, and Udupa Committee, to plan and implement integrative medicine techniques, including yoga, within the healthcare system [10]. Since that time, fast-paced advancements in modern medicine have challenged the integration of yoga into the Indian healthcare system, resulting in the implementation of a variety of models. One commonly followed model is the “co-location” of Ayurveda (a system of medicine based on the idea that disease is caused by an imbalance or stress in a person's consciousness), Yoga, Unani (based on the principle that each individual is viewed as a separate entity, and each factor that makes up an individual is taken into account), Siddha (emphasizes the balance of the five elements of nature within the body), and Homeopathy (based on the theory that treats diseases using the substance that causes the symptoms of a disease in healthy individuals to cure similar symptoms in sick people) [11] (AYUSH) systems, which combine yoga with conventional medical treatment or vice versa. Other models focus on offering yoga as a standalone therapy [12]. Yet another model emphasizes mutual cross-talk between consultants of different medical departments and patients' active involvement in decision-making, known as “systemic integration.”

The National Institute of Mental Health and Neurosciences (NIMHANS), located in Bangalore, India, is dedicated to research, treatment, and training in the fields of mental health and neurosciences. In 2014, the NIMHANS Integrated Centre for Yoga was founded within the Department of Psychiatry to develop and validate specific yoga modules for various neuropsychiatric disorders and conduct clinical trials and mechanistic studies [13,14]. In 2019, the NIMHANS divisions for biomedicine, yoga, and ayurveda were combined into the Department of Integrative Medicine (IMD). The yoga center team offers inpatient and outpatient services. It comprises yoga faculty, yoga therapists (researchers with M.Sc. and/or Ph.D. degrees in Yoga), and yoga scientific officers trained to deliver clinical services such as one-on-one yoga sessions under the supervision of the yoga faculty. The team screens patients for medical conditions and then curates specific yoga modules/practices based on the diagnosis [1].

The advantage of this yoga model is that it can help patients reconnect with their inner selves and begin the recovery process from the ground up, potentially revealing the fundamental triggers for their illness. Positive outcomes from the prototype model have led departments such as Endocrinology, Cardiology, Neurology, Oncology, Obstetrics and Gynecology, Pediatrics, Pulmonology, Psychiatry, and Orthopedics to curate similar models in their respective departments [15,16]. A considerable downside of the NIMHANS model is that it serves as a tertiary care hospital, making it difficult to replicate accurately in other healthcare systems.

Two potential areas of growth for this model have been identified: pluralistic medical education and yoga research. The National Education Policy of 2020 acknowledges the importance of pluralistic medical education, which is the integration of diverse healthcare perspectives in medical education. It mandates that all students of allopathic medical education have a basic understanding of AYUSH. The National Medical Commission has permitted an elective posting in yoga as part of medical internships, and the inclusion of yoga in undergraduate medical education curricula is being considered [13,15]. Textbooks on clinical uses and applications of yoga are already widely available. Several hospitals use yoga to treat non-communicable and psychiatric disorders, and the Indian Psychiatric Society (IPS) recognizes its application in the latter [13]. The prominent players in supporting yoga research are funding agencies such as the Science and Technology of Yoga and Meditation (SATYAM) of the Department of Science and Technology and the All India Institute of Medical Sciences (AIIMS) [1]. Additionally, when considering further research focuses, the indications and contraindications of various yogic practices in different medical conditions, lack of awareness, time constraints, and financial limitations for the patients should be investigated [1].

Public perception of yoga in India

**Figure 1:**
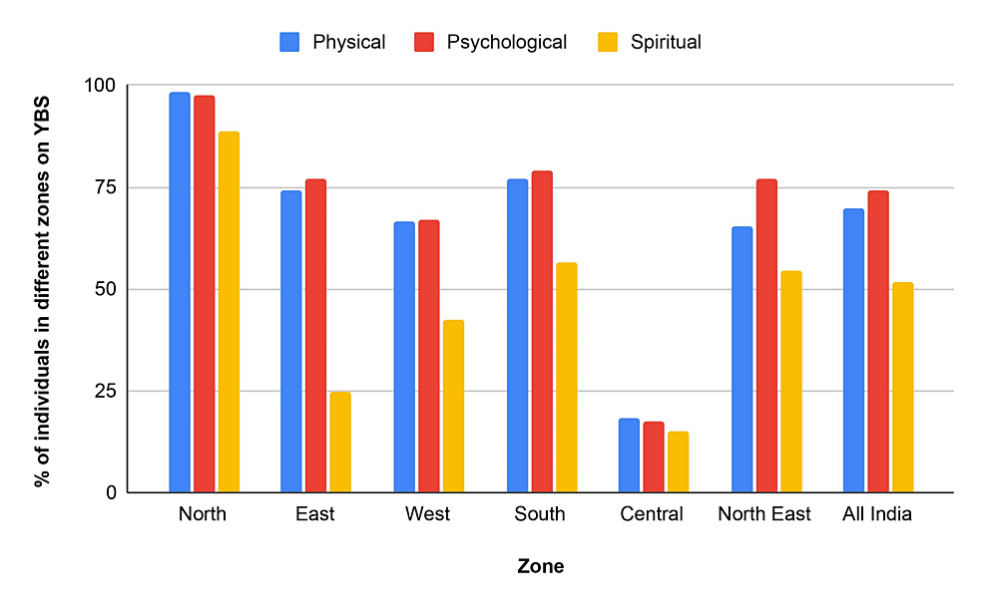
Perception of yoga benefits in different zones of India YBS: Yoga Benefit Scale

Utilizing the validated 18 components of the Yoga Benefit Scale (YBS) and the 19 components of the Barrier for Yoga Scale (BFYS), a 2020 study [12] focused on the perception of the benefits and barriers of yoga in rural and urban India by categorizing the components under physical, psychological, and spiritual well-being rankings. More than 95% of participants agreed that physical fitness is a prime benefit of yoga. Other benefits identified by respondents included increased muscle strength, stress relief, and improved mental alertness. The study found that the benefits of yoga are perceived differently by individuals over 45 years. Those over 45 found present-moment awareness (a practice in which someone focuses attentively on what is occurring in the current moment, including a host of sensations like actions, feelings, and experiences) [17], stamina, and breathing rate more critical, while those under 45 valued higher power and breathing rate. Figure 1 shows the perception of yoga benefits in different zones of India, emphasizing physical, psychological, and spiritual well-being rankings. The North region consistently ranked highest in all three categories, and the national average is included for comparison [12].

**Figure 2:**
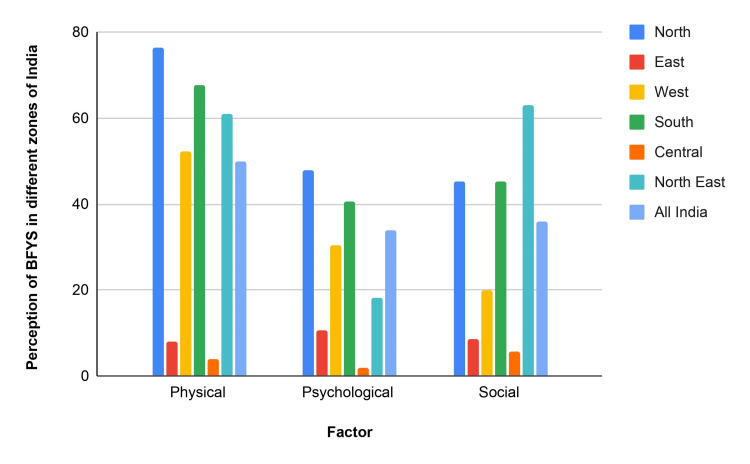
Perception of BFYS in different zones of India BFYS: Barrier for Yoga Scale

The survey revealed multiple obstacles to practicing yoga. Restricted access to facilities and uncertainty about results were the two biggest obstacles for the younger participants. Family commitments were also recognized as a significant barrier, with those over 45 reporting this obstacle as more critical. The other reported barriers were pre-existing medical issues, absent-mindedness, costs, exhaustion, and procrastination. However, the survey did indicate that many participants did not perceive any significant obstacles to practicing yoga, showing a generally positive attitude toward the discipline [12]. Figure 2 shows perceptions of barriers to yoga practice in various regions of India. Physical barriers are the most prevalent in the North, followed by psychological and social barriers. The East has the lowest perception of barriers. The West demonstrates moderate barriers in all categories. The North East is distinguished by its elevated physical, psychological, and social obstacles. Overall, physical, psychological, and social barriers exist for yoga practitioners in India [12].

The role of yoga in promoting physical and mental health

Yoga aims to align an individual's biorhythm with nature. An example is the sleep-wake cycle with the lunar cycle and the rising and setting of the sun. This aims to create an equilibrium between various lifestyle factors such as physical activity, sleep, diet, psychological stress, and social connectivity. This impacts the body on a cellular and molecular level as with the improvement in both cardinal and metabotropic biomarkers of cellular aging [18-20]. Within the past few years, there has been renewed interest in yoga as a popular lifestyle modification method due to the growing recognition of the importance of holistic approaches to health and well-being [20]. Incorporating yoga into mainstream medical practice is a crucial step toward promoting it to the general population. A meta-analysis of 238 studies found yoga has numerous benefits, including improvements in immunological health, mood, pain, anxiety, and auditory hallucinations [21].

Physical Health

Pranayama, an integral component of yoga, originates from the Sanskrit words “prana,” meaning breath of life/vital energy, and “ayama,” meaning expansion/regulation/control [22]. The three types of pranayama are abdominal, diaphragmatic, and thoracic [23], and involve deliberate modifications of the breathing process [24]. Individuals performing pranayama and yogic breathing, which is a fundamental way of breathing in yoga practice by focusing on deepening and expanding the breath for long periods, can observe improved pulmonary function [25]. These improvements are influenced by various factors such as neurocognitive abilities, autonomic and pulmonary operations, as well as biochemical and metabolic activities [26].

Practicing pranayama along with asanas, defined as physical postures or poses practiced in yoga, can help individuals deliberately control the respiratory process [27]. Yogic breathing exercises have been found to significantly improve forced vital capacity (FVC), forced expiratory volume in one second (FEV1), and peak expiratory flow rate (PEFR). The underlying mechanism is the reduction of sympathetic activity, allowing for bronchodilation by correcting abnormal breathing patterns and reducing muscle tone of inspiratory and expiratory muscles, effectively improving the tone and vital capacity of the lungs [28]. Focusing on the abdominal pranayama techniques, Table 1 provides an examination of specific pranayama practices and their associated benefits for individuals with bronchial asthma. Table 1 presents the method of practice (Kapalabhati (KPB) or Bharmari), the specific methods used within the studies, and the results observed, such as improvements in FEV1, FVC, and PEFR, and the potential effectiveness of these pranayama techniques as adjunctive therapies for managing bronchial asthma.

**Table 1:** Impact of yoga on bronchial asthma KPB: Kapalabhati, FVC: Forced Vital Capacity, FEV1: Forced Expiratory Volume in One Second, PEFR: Peak Expiratory Flow Rate

Pranayama Practice	Method of practice	Methods within the study	Results of the study
Kapalabhati	Sitting with the back and neck erect, one should inhale through both nostrils and exhale rapidly by flapping the abdomen during each exhalation at a pace of 60–120 breaths/min [26].	On the day of assessment, experimental group practiced KPB, while control group practiced deep breathing for 10 minutes. Spirometry was conducted to assess FEV1, FVC, and FEV1/FVC ratio before and after the practice [29].	10 min of the practice of KPB enhances FEV1, FVC, and FEV1/FVC ratio in patients with mild to moderate asthma when compared to those practicing deep breathing alone [29].
Bhramari	After a full inhalation, closing the ears using the index fingers, one should exhale, making a soft humming sound similar to a female honeybee [26].	This 12-week study involving 50 bronchial asthma patients (FEV1 > 70%) allocated patients to two groups: group A received 20 minutes of breathing exercises (including high-pitch Omkara). In contrast, group B received 20 minutes of meditation, with subjective assessment, FEV1%, and PEFR measured initially and after 12 weeks [30].	A combination of slow breathing, Bhramari, and Omkara significantly improved symptoms, FEV1, and PEFR in group A patients compared to group B [30].

Yogic practices stimulate parasympathetic activity, lung function, and cellar machinery, causing an increase in maximal oxygen consumption (VO2 max) in both men and women. Furthermore, there is evidence of a generalized decrease in vascular tone [31,32]. Table 2 shows the impact of yoga on various cardiac conditions.

**Table 2:** Impact of yoga on various cardiac conditions HR: Heart Rate, BMI: Body Mass Index, LDL: Low-Density Lipoprotein

Heart Condition	Impact of Yoga
Cardiac Autonomic Dysfunction	Increases HR variability. Increases vagal output. Decreases sympathetic arousal [33].
Arrhythmias	Decreases arrhythmias by reducing sympathetic nervous system activity and promoting parasympathetic output. Decreases the number of symptomatic and asymptomatic atrial fibrillation episodes [34].
Coronary Artery Disease	Decreases BMI, systolic blood pressure, diastolic blood pressure, HR, total cholesterol, triglycerides, and LDL [35].
Heart Failure (HF)	Decreases HR and blood pressure [36]. Improves balance, strength, and endurance [37]. The addition of yoga therapy to standard medical therapy showed improvement in left ventricular ejection fraction [38].

Yoga has been shown to provide various benefits to the musculoskeletal system, such as increasing muscle strength, improving hand grip strength and muscle dexterity, decreasing lower back pain, delaying the onset of muscle soreness, increasing flexibility and balance, decreasing chronic pain and muscle atrophy, increasing motor function and strength, and use of expiratory muscles [39-45]. A proposed mechanism of action for yoga on the musculoskeletal system is an improvement in the bioavailability of nitric oxide and a reduction in catecholamine and angiotensin II levels [46,47].

Yoga asanas reduce muscular spasms, increase flexibility, and reduce body weight [45,48,49]. Studies have shown that 100 conventional abdominal crunches are proportional to a 20-breath yoga breathing workout session due to their effect on the rectus abdominis and external oblique muscles [42]. Additionally, a study conducted on college athletes demonstrated that yoga breathing exercises, as shown by electromyogram (EMG) studies, can enhance muscle utilization by 41%, compared to only 24% with regular training such as flexibility exercises, strength training, skill-specific drills, and participating in the sport itself. Moreover, yoga is advised for older people as it can assist in sarcopenia, improve functional performance, increase bone mineral density (BMD), and reduce the risk of falls [50-52]. Table 3 shows the impact of yoga on the musculoskeletal system.

**Table 3:** Impact of yoga on the musculoskeletal system

Disorder	Yogic Practices	Outcome
Muscular Dystrophy	Sakthivikasaka (Equivalent to completing a session of moderate-intensity exercise)	Increases flexibility and improves muscle strength, tone, and joint stiffness [53]. Decreases blood pressure and improves blood supply to the muscles [46].
Rheumatoid Arthritis/ Osteoarthritis	Land-based exercise programs	Improves arthritis-related pain [54-56]. Decreases erythrocyte sediment rate (ESR) and improves disease activity scores [39].
Spinal Cord Injury	Iyengar yoga	Develops strength, stability, stamina, concentration, and body alignment [57].
Chronic Back Pain	Yoga program as adjuvant therapy	Marked improvements in back-related disability in patients with chronic back pain [43].
Elderly Population	Bi-weekly yoga sessions	Improves physical fitness, muscle strength, power, endurance, and flexibility [58,59].

Mental Health

Studies have shown that yoga can benefit patients with psychiatric conditions by providing symptomatic relief [60] through neurotrophic and psychological factors [61]. The commonly targeted psychological factors are self-regulation, self-efficacy, interoception, embodiment, motivation, connection, self-compassion, psychological flexibility, positive affect, and mindfulness [60-71]. These factors are also targeted by yoga when treating anxiety disorders [72,73] and depression [73]. Table 4 shows the impact of yoga on mental health.

**Table 4:** Impact of yoga on mental health

Intervention	Outcome
Hatha yoga practice	Higher mindfulness scores and significantly lower stress scores in advanced practitioners [74].
Bikram yoga (26 asanas that take place in a heated room)	Improvement in mindfulness and perceived stress [75].
Iyengar yoga with back bends, standing poses, forward bends, and inversions	Improvement in perceived stress. Decrease in state anxiety and trait anxiety. Improvement in depression, Quality of life, and mood states [76].
Kripalu yoga comprising of postures, breathing, relaxation, and meditation	Decrease in total mood disturbance. Decrease in tension anxiety [77].
Yoga group: two yoga classes a week plus two physical exercise classes x six months Control group: only physical exercises (four classes a week) x six months	Significantly lower scores for depressive symptoms. Decrease in scores of anxiety [78].
Sensory-enhanced hatha yoga comprising of asanas, pranayama, relaxation & meditation	Decrease in both state and trait anxiety [79].

Yoga-based asanas are also an effective therapeutic measure for treating depression [80]. Studies have shown that depression scores and biomarkers such as event-related potential (P300) and brain-derived neurotrophic factor (BDNF) decrease and improve after yoga therapy. Notably, a positive BDNF response is also seen in the therapeutic effects of antidepressant drugs and electroconvulsive treatment [81]. Additionally, cortisol, a biochemical associated with depression, is reduced by yoga therapy and helps alleviate the overactivity of the hypothalamic-pituitary-adrenal (HPA) axis [82]. Moreover, active yoga has a mood-stabilizing effect and increases gamma-aminobutyric acid (GABA) levels [83]. Chanting the “OM” mantra during yoga has been found to exert effects on areas of the brain associated with emotions, potentially through vagal afferent stimulation [84]. Numerous research papers have consistently supported the effectiveness of yogic interventions such as Sahaj Yoga for depression [85-89].

Combining yoga with cognitive behavioral therapy (CBT) has been recognized as a complementary therapy for psychiatric conditions. Yoga's relaxing effects have been found to reduce negative cognitive patterns such as rumination while promoting concepts such as awareness of negative thinking [82,90]. Research has demonstrated that therapeutic yoga can enhance and augment certain aspects of CBT in adults with anxiety and depression by significant improvements in emotional self-regulation [82].

Incorporating yoga into the US healthcare system

Yoga has gained worldwide popularity in improving overall health and well-being. In a 2016 survey, 36 million Americans practice yoga regularly [91]. This ancient practice was first brought to the United States by Swami Vivekananda in 1893. The significance of yoga has been recognized globally, and the United Nations declared June 21st as the International Day of Yoga/World Annual Yoga Day in December 2014 [92]. The World Health Organization's Global Action Plan 2018-2030 also recognizes yoga to enhance physical activity [93].

In recent times, there has been increased interest in integrating yoga as a holistic healthcare model in the US due to the evidence-based research supporting its potential physical and mental health benefits, some of which are noted in the previous section. There are several potential areas where yoga could be incorporated into the existing US healthcare framework, including preventive medicine, chronic disease management, behavioral health, and rehabilitation. As yoga's incorporation into the US healthcare system acquires popularity, it is essential to address the challenges and consider evidence-based recommendations for its successful incorporation.

Challenges and recommendations of incorporating yoga into the US healthcare system

Integrating yoga into preventive medicine practices in the United States faces challenges due to insufficient awareness of evidence-based studies in the literature. Studies have identified several barriers to practicing yoga in the US, including the perception that it does not provide enough physical and weight loss benefits, fear of injury, low self-efficacy to perform the practices, preference for other physical activities, and scheduling difficulties [94]. These barriers impede the widespread implementation of yoga as a complementary therapy in the healthcare industry. There are several challenges to integrating yoga into the US healthcare system, although evidence-based recommendations to overcome them do exist.

Fear of Adverse Effects

Although a few studies have reported adverse effects of yoga, it is essential to note that proper guidance and supervision can substantially reduce the risk of adverse side effects [95,96]. In addition, pranayama is generally considered safe, with only rare reported adverse effects, including spontaneous pneumothorax, rectus sheath contusion, and pneumomediastinum [97,98]. The perception that yoga lacks physical and weight loss benefits, fear of injury, low self-efficacy, preference for other physical activities, and scheduling difficulties [94] are also significant barriers to yoga practice. Overcoming these obstacles requires providing education and information on yoga's physical and mental benefits. Additionally, offering beginner-level classes and modifications for varying ability levels can increase self-efficacy and decrease injury anxiety.

Lack of Standardized Protocols

A significant barrier to integrating yoga as a complementary therapy is the absence of customized yoga therapy referrals, such as those written for physical or occupational therapy [99]. To overcome this barrier, it is essential to promote the inclusion of yoga into medical and mental health treatment protocols and educate healthcare professionals about its potential benefits. Developing evidence-based, standardized yoga prescription guidelines that tailor to the needs of different populations can improve accessibility and enable the integration of yoga therapy into regular healthcare practices. This may also be a key step toward health insurance coverage for yoga classes.

Cost of Yoga Classes

The cost of yoga classes may also be a significant obstacle to participation, especially for those with lower incomes [100]. To overcome this barrier, one option is to provide subsidies or reimbursement programs for yoga classes as part of health insurance plans. Yoga classes can also be offered at minimal to no cost in community centers, colleges, and other public spaces.

Lack of Perceived Value for Integrative Medicine

Another significant barrier is the lack of respect for integrative medicine in the United States [101]. Many healthcare professionals may not consider complementary therapies such as yoga a legitimate treatment option. To overcome this barrier, educating healthcare providers on the benefits of integrative medicine and highlighting the existing research supporting its efficacy is essential. Incorporating integrative medicine curricula in medical schools may also increase respect for its potential benefits. This would provide future physicians with the necessary skills and knowledge to offer yoga to their patients as a safe and effective adjuvant treatment option.

Time Constraints for Patients

The work culture in the US presents a significant barrier to yoga, as patients often struggle to find time to attend regular classes due to demanding work schedules and other responsibilities [94]. To surmount this challenge, healthcare providers can play a vital role in supporting patients by offering online resources such as instructional videos, guided meditation sessions, and yoga apps. Virtual yoga classes conducted through video conferencing platforms offer flexibility, real-time instructor guidance, and a sense of community, enabling individuals to practice yoga conveniently from home or during work breaks, empowering patients to integrate yoga into their daily lives as a valuable self-care activity despite the challenges of the US work culture.

CHallenges With Policymakers

In addition to healthcare providers and patients, policymakers also face significant obstacles in incorporating yoga into the US healthcare system. These obstacles stem from a lack of a clear definition of yoga, a lack of awareness about evidence-based research supporting yoga's benefits, cost-effectiveness, and difficulty with long-term adherence [102]. To overcome these barriers, clear and standardized definitions of yoga have to be established. Policymakers must spread awareness of the evidence-based benefits of yoga and evaluate cost-effectiveness. They must support long-term adherence initiatives and incorporate yoga into public health policies by engaging stakeholders and advocating for integrative medicine.

Action plan

A multi-faceted approach would be necessary to further the implementation and integration of yoga within the US healthcare system. Initiatives, such as NIMHANS implemented by the Indian healthcare system, may promote the potential physical and mental health benefits of yoga and its integration into people's daily routines [103]. It is crucial to acknowledge the potential limitations and challenges associated with integration to ensure effective implementation. One of the primary challenges is the perception that yoga does not provide enough physical and weight loss benefits discouraging people from engaging in regular practice. Moreover, the cost of yoga classes may pose a significant obstacle for individuals with lower incomes, ultimately limiting access to those who need it the most. More limitations include the lack of standardized protocols for yoga therapy, fear of injury, and scheduling difficulties due to time constraints. Healthcare providers must embrace yoga as an adjunct therapy for respiratory, cardiac, musculoskeletal, and psychiatric disorders. Incorporating yoga into school curricula, workplaces, and community centers and establishing community-level programs for yoga educators and leaders may aid in promoting the practice of yoga. To guarantee the quality and standardization of yoga practice, institutes of excellence, such as those in India, may support high-quality research regarding yoga and the development of treatment protocols.

Policymakers play a vital role in this action plan by spreading awareness about the potential benefits of yoga, evaluating the cost-effectiveness of incorporating yoga into US healthcare systems and supporting policies that advocate for integrative medicine and its inclusion in mainstream healthcare practices. They can allocate funding for research supporting clinical trials to gather robust evidence. They can work towards integrating training about yoga into medical school curricula to make future physicians knowledgeable about incorporating yoga as part of patient treatment plans. Moreover, they can collaborate with relevant stakeholders to establish evidence-based guidelines and standardize yoga therapies. Lastly, policymakers can advocate for insurance coverage for yoga classes as a reimbursable and accessible healthcare service for all.

## Conclusions

Yoga is an integral element of India's healthcare system and is profoundly rooted in its culture. It has evolved over time, integrating numerous forms, mental discipline, controlled postures, conscious breathing, detachment, and meditation. Integrating yoga into the US healthcare system, modeled by the Indian system, could prove valuable in enhancing the overall physical and emotional health of the population. Despite the potential benefits, incorporating yoga into the US healthcare system has various complex economic, social, and systemic challenges that must be addressed. To tackle these challenges, evidence-based recommendations such as beginner-level classes and modifications, standardized yoga therapy guidelines, and insurance coverage for classes may be effective. In addition, policymakers and healthcare professionals must also play a pivotal role in integrating yoga into wellness programs, schools, and fitness centers. Furthermore, regulatory bodies and centers of excellence must be established to ensure the standardization and quality of yoga practice. International actors such as the UN have taken the first step to acknowledge yoga on an international platform by creating an observance day for yoga. Further steps need to be taken by the UN and other key international organizations to cultivate a global dialogue and promote the use of integrative medicine throughout the world's healthcare systems.
